# Applicability of a digital health application for cancer patients: a qualitative non-participation analysis

**DOI:** 10.1186/s12913-024-11654-0

**Published:** 2024-10-05

**Authors:** Klara Pfeifer, Mitra Tewes, Stefan Kasper, Jörg Hense, Jan Franco, Martin Schuler, Christoph Schöbel, Gülay Ates

**Affiliations:** 1 Department of Palliative Medicine, University Hospital Essen, University of Duisburg-Essen, 45147 Essen, Germany; 2 Department of Medical Oncology, University Hospital Essen, West German Cancer Center, University of Duisburg-Essen, 45147 Essen, Germany; 3 German Cancer Consortium, Partner Site University Hospital Essen, 45147 Essen, Germany; 4 Center for Sleep- and Telemedicine, West German Lung Center at University Hospital Essen, Ruhrlandklinik, 45239 Essen, Germany; 5 Institute for Digitalization and General Medicine, University Hospital Aachen, 52074 Aachen, Germany

**Keywords:** Digital therapeutics, Digital health application, Cancer disease, Insomnia, Barriers, Interviews

## Abstract

**Background:**

The use of digital health applications (German acronym DiGA) for comprehensive patient care is increasing rapidly. Patients with non-organic insomnia can be prescribed an application to manage insomnia. Due to the high prevalence of insomnia in patients with cancer, we were interested in the effect of it and what barriers need to be overcome for its use. The focus of existing studies on acceptance and benefits prompted us to emphasise the analysis of barriers and thus to formulate possible solutions.

**Methods:**

To analyse the barriers of use, the study population (patients with self-reported tiredness or sleep disturbance via validated instruments and cancer disease) was divided into 3 groups. In groups 1 (patients who refused to participate in advance) and 2 (patients who refused a prescription), short close-ended questionnaires were used for non-response assessment by treating oncologists. Problem-centred guidelines were used for the telephone interviews with group 3 (patients who did not provide information on DiGA use). Alternatively, group 3 was invited to complete and return the close-ended questionnaire. A quantitative analysis of the non-response reasons was conducted using SPSS in groups 1 and 2, while MAXQDA was used for the qualitative data in group 3.

**Results:**

Patients refused to participate at several stages of our study. Quantitative data are available for groups 1 and 2. In the largest group 1, 62% of patients refused to participate due to non-subjective sleep disturbance (177 out of 189 patients) during recruitment by treating oncologists, despite high scores on the screening tool. In the small group 2 (11 out of 15), the most common reasons for withdrawal documented by the oncologists were loss of interest and deteriorating health. The problem-centred qualitative interviews with group 3 (17 patients) revealed that some of them used the prescribed DiGA, despite not being included in the main study and being categorized as lost to follow-up.

**Conclusion:**

Analysis of barriers to DiGA use showed that reducing administrative barriers and providing digital and personal support can increase acceptance of the use of DiGAs among cancer patients. Additionally, screening tools can act as a door opener to further communication regarding DiGAs.

**Trial registration:**

German Register of Clinical Trials DRKS00034198, registration date: 7/05/24 (retrospectively registered).

**Supplementary Information:**

The online version contains supplementary material available at 10.1186/s12913-024-11654-0.

## Background

Since December 2019 the German Digital Healthcare Act (DVG) came into force, digital health applications were included in directory of reimbursable DiGAs [1]. A fast-track procedure has been established for the rapid implementation of these low-risk medical devices [2]. This process entails rigorous assessment of safety, functional suitability, and medical benefits [1–3]. Once approval has been granted by the German Federal Ministry for Drugs and Medical Devices (BfArM) and inclusion in the DiGA directory has taken place, a prescription can be provided by physicians or physiotherapists. Alternatively, direct authorisation may be granted by the relevant health insurance company. Subsequently, the health insurance company generates the required activation code for the patient’s end device [4, 5].

The use of digital health applications (German acronym DiGA) is increasingly finding its way into clinical practice, as they are designed to support patients’ individual disease management [3]. Advances in digital communication and the growing presence of various start-up companies in the German healthcare market mean that the range of prescription ready DiGAs is becoming increasingly broad and extends across different specialisms [6]. In the period from 2020 to 2022, 203,000 DiGAs were prescribed in Germany. 164,000 (81%) were redeemed. With 16,000 prescriptions for insomnia patients, it is one of the 4th most frequently prescribed DiGAs. The proportion of prescriptions authorised by health insurance companies is 12%, with 90% first prescriptions and 10% second or third prescriptions. It is represented and used by both genders in all age groups, with women consistently receiving more prescriptions than men. What all DiGAs have in common is that the number of follow-up prescriptions after a first prescription decreases [7]. According to the German Association for Digital Healthcare, Germany is playing a pioneering role in the field of DiGA. Other European countries have adopted a similar approach, utilizing a fast-track procedure. France, for example, has been using the PECAN model since 2021. Initiatives to establish a system of harmonised approvals are also underway at European level [8, 9].

There are currently 64 applications listed in the Germany DiGAs directory, divided into 11 organ-related categories and one ‘other’ category [10]. In 2024, 35 of the 64 DiGAs are permanently listed in this directory. One of them is ‘somnio’^®^ (https://somn.io/), which was approved in October 2020 for the treatment of insomnia. The application is aimed at patients suffering from insomnia symptoms and works with evidence-based methods from the field of cognitive behavioural therapy [11]. According to the guideline of the German Society for Sleep Research and Sleep Medicine, these techniques continue to be the gold standard for the treatment of sleep disorders [12].

‘somnio’^®^ guides its users through the various modules by ‘Albert’, a virtual sleep therapist. Information is conveyed in an easy-to-understand language and the speed of speech can be adjusted if necessary. The effectiveness of the application was proven in a randomized controlled study with 56 participants. The intervention group reported that their time spent asleep and awake was shortened and the total sleep time was increased, with 56% of participants confirming this. The DiGA ‘somnio’^®^ meets the necessary requirements for inclusion in the permanent DiGA directory, as evidenced by repeated positive intervention-control-group studies [11, 13].

The high prevalence of insomnia as a concomitant symptom of cancer diseases now gives rise to the question of whether cancer patients can also benefit from a prescription and achieve an improvement in sleep-related parameters. As the literature research showed, sleep disorders and the resulting effects on quality of life in everyday life are common side effects of various cancer diseases [14, 15]. The importance of early intervention was emphasised as early as 2001 in a study by Savard et al., which looked at the relationship between cancer and insomnia [16]. As meta-analyses have shown, the cognitive behavioural therapy techniques used within the DiGA are the treatment of choice for comorbid insomnia associated with cancer diseases [17, 18].

The DiGA could therefore also be a suitable tool for improving insomnia symptoms in cancer patients.

The area of DiGAs in the medical care system is undergoing rapid development. Nevertheless, there are indications that scientific evaluation is encountering limitations. In the study conducted by Labinsky et al., it was concluded that there is a lack of empirical data concerning DiGAs, and thus, the implementation of larger-scale practical studies was advocated. A frequently observed phenomenon is unit non-response and high drop-out rates in the intervention group in comparison to the control group. Further investigation into drop-out analysis would serve to enhance the methodological quality of various DiGAs, as well as the validity and reliability of the findings [19, 20].

A usage analysis relating exclusively to DiGAs was carried out by a German health insurance in 2021. For this purpose, 244 insured persons with a DiGA prescription were surveyed. 62% found the DiGA helpful and around half would continue to use it in the future. In contrast, 33% were dissatisfied, with a lack of added value and a lack of individualisation cited as the main reasons [21].

Our literature search revealed a clear focus of the existing studies on the acceptance and benefits of DiGAs [19, 22, 23] whereas non-response analyses on practicability and barriers have hardly been the subject of previous research [24, 25]. It is of particular importance to consider the various challenges faced by vulnerable groups when attempting to access digital resources. In order to facilitate equitable access for all target groups, including patients with chronical illnesses or with continuing health impairments, it is essential to gain a comprehensive understanding of the barriers and obstacles that may be encountered outside of digital literacy, in order to ensure that all members of society have the opportunity to engage with DiGAs [26]. A review of the literature published from January 2016 to December 2021 on mobile phone-based interventions reveals, on the one hand, the benefits of mHealth for cancer patients and, on the other hand, the lack of research into the challenges and obstacles of non-utilisation despite an existing prescription [27]. The findings of a cross-sectional survey on e-mental health interventions for psychiatric patients by Weitz et al., published in 2023, also emphasise the potential and importance of tailored implementation strategies to improve healthcare [28].

Given the critical importance of dropout rates to the validity and reliability of trials, it is essential to investigate the reasons for dropout at different stages of a main trial. This will help to identify ways and strategies to address the barriers to the use of DiGAs as a means of patient empowerment. Furthermore, the barriers known in the literature, viewed from the perspective of equity of access, should contribute to a more comprehensive understanding of the groups affected and to the formulation of potential solutions [29].

As with other DiGA studies, a non-negligible number of patients did not receive a prescription or did not provide information on their DiGA activation or use within our main ‘SOMNUS’-study (benefit of SOMNio in cancer patients-analysis of qUality of life and phySical activity). Consequently, we systematically and partially standardised the reasons for non-use from the patients’ perspective in our study. Additionally, the reasons for the refusal of the treating physicians to prescribe the DiGA were documented. All barriers mentioned are the main objectives of the non-participation analysis presented in this paper.

## Methods

### Study population of the main study

The outpatient clinic of a German comprehensive cancer center routinely performs a tablet-based patient reported outcome measurement for cancer patients. This routine data was used to identify patients who provided information on tiredness and/or sleep disturbances in a symptom and needs assessment in the period January 2021-May 2022. The minimal documentation system (MIDOS^2^) is the validated German version of the Edmonton Symptom Assessment Scale for assessing the symptom burden of oncology patients and was used to record tiredness. The Patient-Health-Questionnaire (PHQ-8), a validated instrument for the assessment of depressive disorders, provided an initial evaluation of sleep disturbance [30–32]. The inclusion criteria for the main ‘SOMNUS’-study were initially adult patients with histologically confirmed cancer. Then patients are systematically screened in a symptom and needs assessment and included if an adult patient reports as a second important criterion: ‘I have difficulty falling asleep or sleeping through the night, an increased need for sleep more than half of the days or almost every day’ or reports moderate or severe tiredness and weakness. The Declaration of Helsinki and Fortaleza will be adhered to in all steps and upon participation and patient consent, a further baseline assessment was performed by an oncologist using the Insomnia Severity Index (ISI). For the categorisation of insomnia, the threshold values for insomnia classification by Dieck et al. were adopted: 0–14 points: no/subthreshold insomnia, 15–21 points: moderate insomnia, 22–28 points: severe insomnia [33].

Based on these criteria, adult patients with an ISI score of at least 15 were recruited and will only be included as a case in the main study analyses if further criteria are met. Firstly, they had to provide feedback on DiGA activation and secondly, they had to return at least two of the three follow-up questionnaires in order to be included as a case in the main ‘SOMNUS’-study.

### Study population of non-participation and dropout surveys

If an ISI of at least 15 points was achieved, the DiGA could be prescribed by the treating oncologist in the case of moderate insomnia. In several phases of our main ‘SOMNUS’- study, eligible participants refused to take part in a trial of a DiGA for the treatment of sleep disorders. Accordingly, different definitions of non-participants were defined for each phase, as follows:

1. Patients who refused to participate in the study in advance (unit non-response),
2. Patients who refused a prescription (drop-out),
3. Patients who did not provide any information on DiGA use (lost-to-follow-up).

As can be seen in Table 1, group 1 consists of people who met the above-mentioned criteria for study participation and who declined to participate in the study in advance (anonymised evaluation of patient records). The second and third group contain patients who also belong to the study population, agreed to participate in the study and completed the first screening survey (1st questionnaire) including ISI questions, but did not participate at different times thereafter.

Group 2 therefore consists of participants who signed a consent form to participate, but for whom the patient himself or the treating physician then refrained from prescribing, which would have been the next important step for the main ‘SOMNUS’-study. Finally, group 3 is made up of participants who were prescribed DiGA without completing the main study questionnaires. The non-participants in group 3 lacked information about their DiGA use and/or positive or negative experiences with use or any challenges including difficulties associated with DiGA (Table 1).

**Table 1:** Grouping according to the response behaviour

Group 1^a^ (unit-non-response)	Group 2 (study drop-out)	Group 3 (lost-to-follow-up)
Patients who refused to participate in advance^a^ The reasons were asked by the treating oncologist and documented the mainly close-ended questionnaire.	Eligible patients (ISI ≥ 15) with informed consent to participate in the study and then drop-out due to no DiGA prescription. The reasons were asked by the treating oncologist and documented at the one page mainly close-ended questionnaire.	Patients who agreed to participate at ISI ≥ 15, received a prescription and subsequently did not reply to main study questionnaires. The reasons were asked by the author and documented in writing. (Telephone call, e-mail, questionnaire, letter, feedback via treating oncologist)

^a^As the anonymised parameters evaluated are based on routinely collected data, no separate patient consent was required for the first survey

### Survey methods

#### Unit non-response using a partially standardised closed external survey

##### Group 1

Reasons for refusal immediately after being informed about the possibility of participating in the study were asked by the treating oncologists and documented using a mainly close-ended questionnaire with the following response options: Patient refused despite positive MIDOS^2^ and/or PHQ-8 questions due to ‘no subjectively perceived sleep disturbance or tiredness’ or another reason noted by the physician. Another level was a medical exclusion, which documented the reasons for positive MIDOS^2^ and/or positive PHQ-8 questions in patients with a ‘life expectancy of less than 6 months or ‘insufficient’ knowledge of German language”.

#### Study drop-out by means of a largely standardised closed external survey

##### Group 2

During the second appointment in the outpatient clinic and after the refusal of the prescription by the physician or patient, the reasons were also documented by the treating oncologist on mainly close-ended questionnaire for the respective patient. The standardized questionnaire provided the following answers to choose from and allowed an open answer option for ‘other reason’.

DiGA not prescribed because:

- no interest.
- no subjective tiredness.
- too stressful next to therapy.
- no access to smartphone/tablet.
- too time-consuming.
- other reason: _________________________________.

#### Lost-to-follow-up qualitative interview guide: Self-disclosure

##### Group 3

Patients who did not provide feedback on DiGA use after receiving a prescription were contacted by an author between January 2022 and March 2022 and asked for personal or written feedback. The primary interest was in face-to-face conversations in person or on the phone. To ensure a more accurate comparison of the responses, a problem-centred interview guide was utilized with a focus on the initial steps and experiences after obtaining a prescription. This was done with the objective of gaining a comprehensive understanding of the potential challenges associated with the activation process and application itself. Therefore, the introductory open question to all telephone interviewees was: ‘*What did you do with the prescription after your appointment?’* Further open alternative or additional questions dealt with the recording of personal circumstances, time and validity period of the prescription, knowledge of the redemption route and the further procedure after receiving an access code as well as during and after DiGA installation. Perceived challenges were asked about in detail in all steps from receipt to installation and use of the DiGA.

If patients could not be reached or expressed the wish to respond in writing, a personalized letter with a partially close-ended multiple response questionnaire was sent by post (including a stamped envelope) or by email if there was no response for a telephone interview. The short questionnaire designed for this purpose contained a multiple-answer set based on the problem-centred interview. The multiple answer selection addressed health status, health insurance, receipt of prescription code, expiry date or validity of prescription, instructions, technical difficulties, lack of contact person for installation, etc. There was also an open response option to enter ‘other reasons’ for feedback in own words.

### Evaluation methods

All data was recorded and saved electronically in order to be able to recognise any regularities in the non-response analysis. Group 1 and 2 physicians’ non-study participation data, mostly close-ended entries, were entered into SPSS. The SPSS (Statistical Package for Social Sciences-Version 29 https://www.ibm.com/de-de/spss) programme was used for the descriptive analyses and measures of central tendency and dispersion. Only valid percentages are reported for physician-documented refusals within group (1) SPSS was also used to analyse data from group (2) However, due to the small sample size in group 2, only participant numbers are reported instead of percentages.

In group 3, the qualitative data based on the transcripts of the face-to-face and telephone interviews and the written material sent in, were entered, and stored electronically for each qualitative interview. The pseudonymized qualitative data material was coded according to the problem-centred guidelines using the content analysis method. On the one hand, it was theory-driven and thus deductive coding, but on the other hand, it was open and thus inductive to extract emerging themes from the material.

To ensure accuracy and quality, four texts by two authors were coded separately using MAXQDA and a coding tree was then developed jointly. In addition to discuss the comparison and optimization of categories and determining the coding tree (see Additional file 3), an intercoder reliability calculation was carried out, which resulted in a kappa value of ‘almost perfect’ [34]. A researcher then coded all responses from group 3 according to the jointly agreed coding tree. To structure the statements, a code system with 6 main codes (redemption at health insurance, withdrawal of study participation, code received, app activation, experiences and usability, interest in follow-up-prescription) and respective subcodes was developed with the help of MAXQDA.

## Results

From the beginning of the study, a close-ended questionnaire was developed, and a systematic documentation process was carried out by the recruiting physicians to ascertain reasons for non-participation (group 1). Once consent had been obtained and the declaration of participation signed, physicians completed a further close-ended questionnaire in order to ascertain the reasons for drop-out at this stage of the study (group 2). At the final stage of the study, which included participants who had consented to participate, signed informed consent forms, received receipts, and did not respond to the main study questionnaires, a non-response analysis was conducted through telephone interviews and/or by mail when requested. Finally, a qualitative problem-centred interview for a lost-to-follow-up analysis was conducted in parallel with the main study to gain insights through narrations (group 3). This phase involved telephone interviews and/or mail correspondence upon request.

### Group 1 (refusal to participate in the study in advance)

Due to the high number of patients who refused to participate in the study in advance, the reasons for refusal within the 1st group of patients who refused to participate in the study were also analysed retrospectively.

**Fig. 1:**
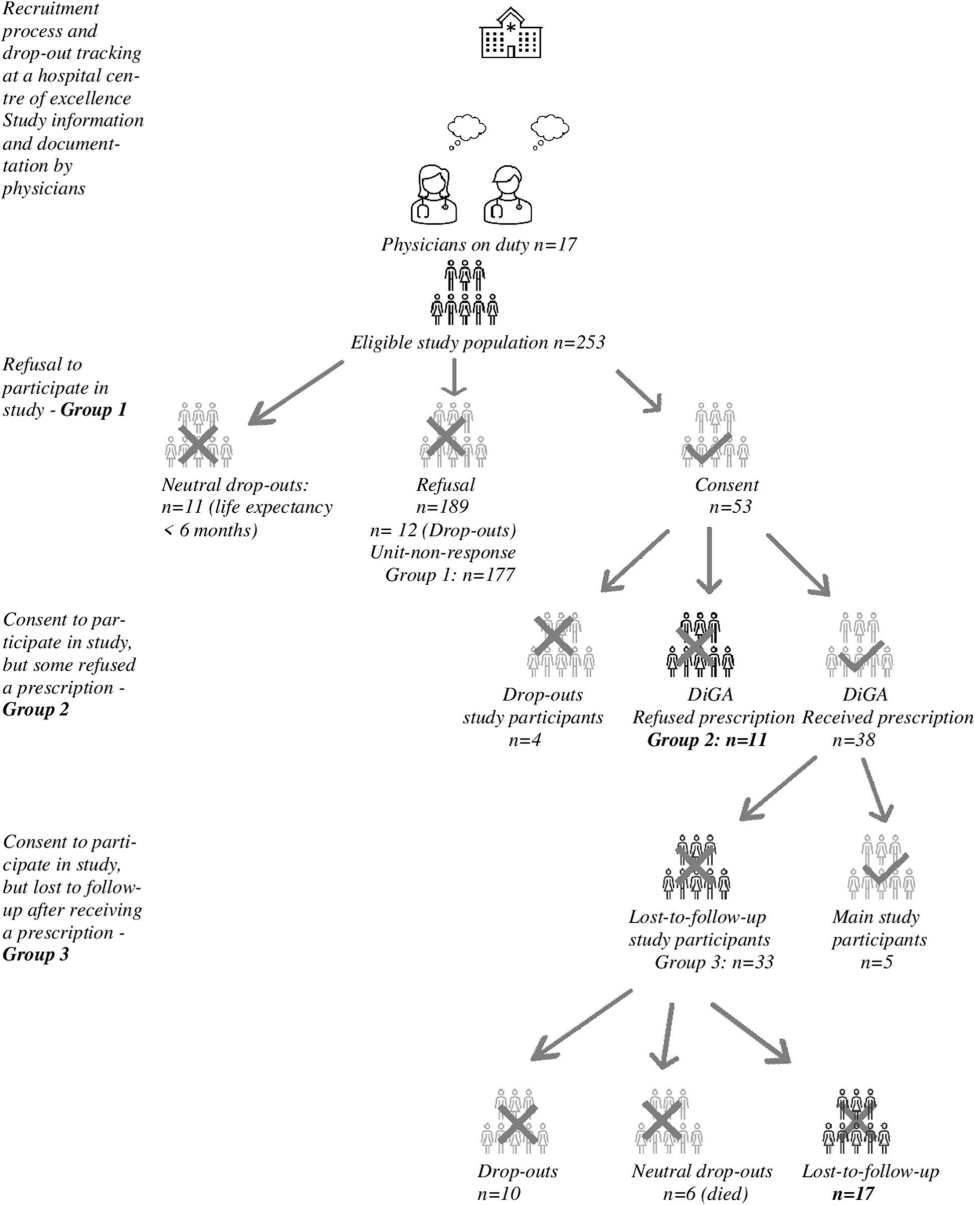
Course of study and DiGA regulation process. Course of study and DiGA regulation process; source: own data, own calculations

In accordance with the above-mentioned inclusion criteria, 253 patients were included in the non-participation analysis by the treating oncologists during the survey period. Of these, 189 patients refused to participate in the study despite positive MIDOS^2^ and/or a positive PHQ-8 question. Within these 189 patients, the oncologists recorded the reasons for non-participation for 177 patients, which corresponds to a response rate of 94% in group 1 (Fig. 1).

The genders in group 1 were almost equally distributed (men: 55%, *n* = 98; women: 45%, *n* = 79). The mean age was 61 years (min. 23, max. 85, standard deviation 12).

The majority of patients suffered from lung cancer (38%) and carcinomas of the gastrointestinal tract (23%). 135 patients were in stage IV according to the Union for International Cancer Control (UICC) at the time of the initial survey. With regard to the use of tranquillisers, antidepressants or sleeping pills, 75% (*n* = 129) of the respondents indicated that they had not used such medications. 17% (*n* = 29) reported daily use and 8% (*n* = 13) reported occasional use (see Additional file 1).

In group 1, 110 out of 177 patients justified their refusal with ‘no subjective sleep disorder’ (response rate: 94%).

This indicates that 62% of patients did not perceive the sleep disorder as such in the subsequent anamnesis interview with the physician, despite reporting sleep disorder or tiredness in a previously utilised standardised assessment tool. Consequently, they also saw no necessity for treatment with a DiGA to enhance sleep behaviour. The other reasons for non-participation are very fragmented and split into many different categories with low frequencies. For example, 10% (17 patients) stated that they were not interested, a total of 9% cited their state of health or psychological stress, 8% a language barrier, followed by 8% other reasons. The lack of technology played a role in only 3% of 177 patients. For one patient, the doctor documented the reason for refusal as follows:

‘(…) *the above-mentioned patient does not take part in the study after approx. two weeks of reflection and detailed study of the information documents for the following reason: He regards the study and the app as a further attempt to further reduce and automate doctor-patient time and communication and thus ultimately to replace the doctor with computers. He does not want to support this…’ (physicians’ documentation of patient’s reason for refusal of study participation)*.

### Group 2 (refused a prescription after agreeing to participate in the study)

A total of 53 patients agreed to participate after being informed about the study. Of these 53, 15 patients did not receive a DiGA prescription. Of these 15, 4 patients could not be contacted after three attempts and are in the ‘drop-out’ category. The remaining 11 patients without a DiGA prescription were asked about their reasons for refusing a prescription; response rate 73% (Fig. 1).

Group 2 consisted of 2 women and 9 men, the median age was 58 years (range 42, min. 30, max. 72). The following cancer entities were represented in descending order: Carcinoma of the gastrointestinal tract (3 patients), lung (2 patients), male genital tract and urinary tract (2 patients), breast (1 patient) and soft tissue (1 patient), unspecified cancer entity (2 patients). 10 patients were in stage IV according to UICC at the time of the initial interview, 1 patient in stage I. The use of sleeping pills was denied by 5 patients, stated as occasional by 3 and daily by 2. One patient did not report this (see Additional file 2).

For all 11 patients, the reasons for refusal were also documented by an oncologist. Three patients lost interest in the meantime and one patient saw no reason to participate in the study due to subjectively not feeling tired. In 7 patients, the current state of health was the reason for refusing a prescription. The disease-related factors mentioned were worsening of the patient’s condition due to cancer progression, concomitant symptoms of the disease and improvement of the sleep disorder after completion of the cancer therapy. Below are 3 examples of the different reasons for refusal:

*‘The patient was transferred to the palliative care ward due to severe progression of his cancer. I refrained from issuing a prescription.’* (*Treating physician about the possibility of a DiGA prescription*).

The patient was mainly suffering from *‘sleep disorders due to his neobladder’* (post-cystectomy for urothelial carcinoma of the bladder, author’s note). No prescription was issued. (*Treating physician about the possibility of a DiGA prescription*)

*‘Mr…is no longer in pain after radiotherapy and thinks that the app no longer makes sense for him. Looking back*,* he sees the sleep problems exclusively as being caused by cancer pain. I wouldn’t prescribe the app at the moment.’* (*Treating physician about the possibility of a DiGA prescription*).

### Group 3 (after consent at ISI ≥ 15 also agreed to a prescription, then non-utilisation)

Of the 38 patients who received a prescription after consent, 5 patients activated the DiGA as defined in the main study and a further 6 died. Of the remaining 27 patients with a prescription, 10 patients could not be reached after being contacted three times. In group 3, the analyses are therefore based on a total of 17 cases and provide an insight into the reasons that led to a lost-to-follow-up case from the main study; response rate of 63% (Fig. 1).

The responses were obtained in different ways in order to reach as many self-reporting patients as possible; 6 patients via guided telephone interviews and 11 via written correspondence. Of these 11 written responses, 5 patients provided reasons by means of a partially standardised questionnaire and 6 patients formulated their reasons for non-use in their own words.

**Fig. 2:**
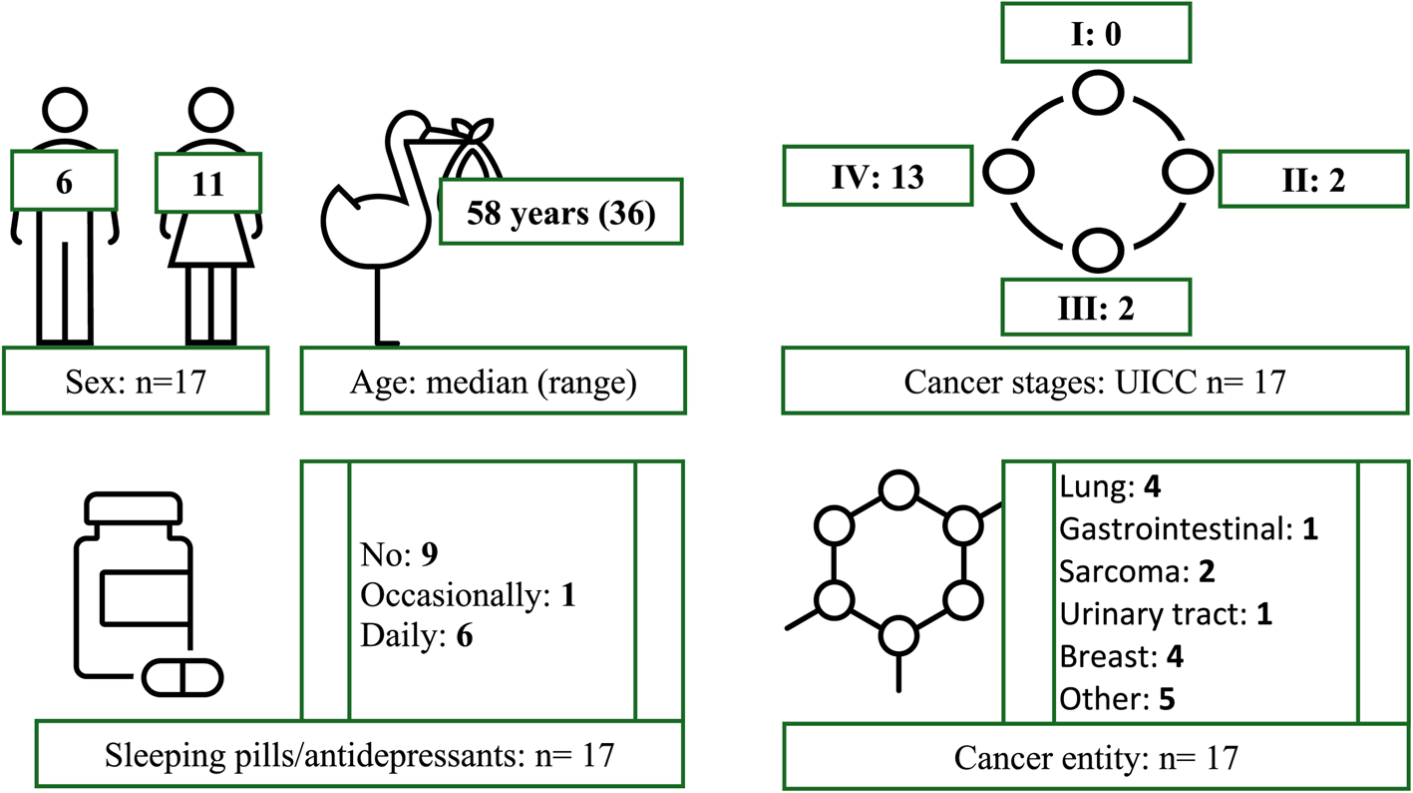
Patient characteristics - Group 3. Source: Document analysis; reporting period: Jan. 2021-May 2022; own calculations

As can be seen in Fig. 2 group 3 (*n* = 17) consisted of 11 women and 6 men, the median age was 58 years (range 36, min. 39, max. 75). Four patients each suffered from breast and lung cancer, two from sarcoma and one patient each from the following entities: cancer of the gastrointestinal tract, head and neck cancer, bone cancer, urinary bladder cancer, cancer of the male and female genital tract, unspecified cancer entity. With 13 patients, the majority were in stage IV according to UICC, 4 patients were in stage II and III. 9 patients denied using sleeping pills, tranquillisers, or antidepressants, 6 stated daily use and 1 patient occasional use. 1 patient made no statement about medication use (Fig. 2).

### From the prescription onwards

**Fig. 3:**
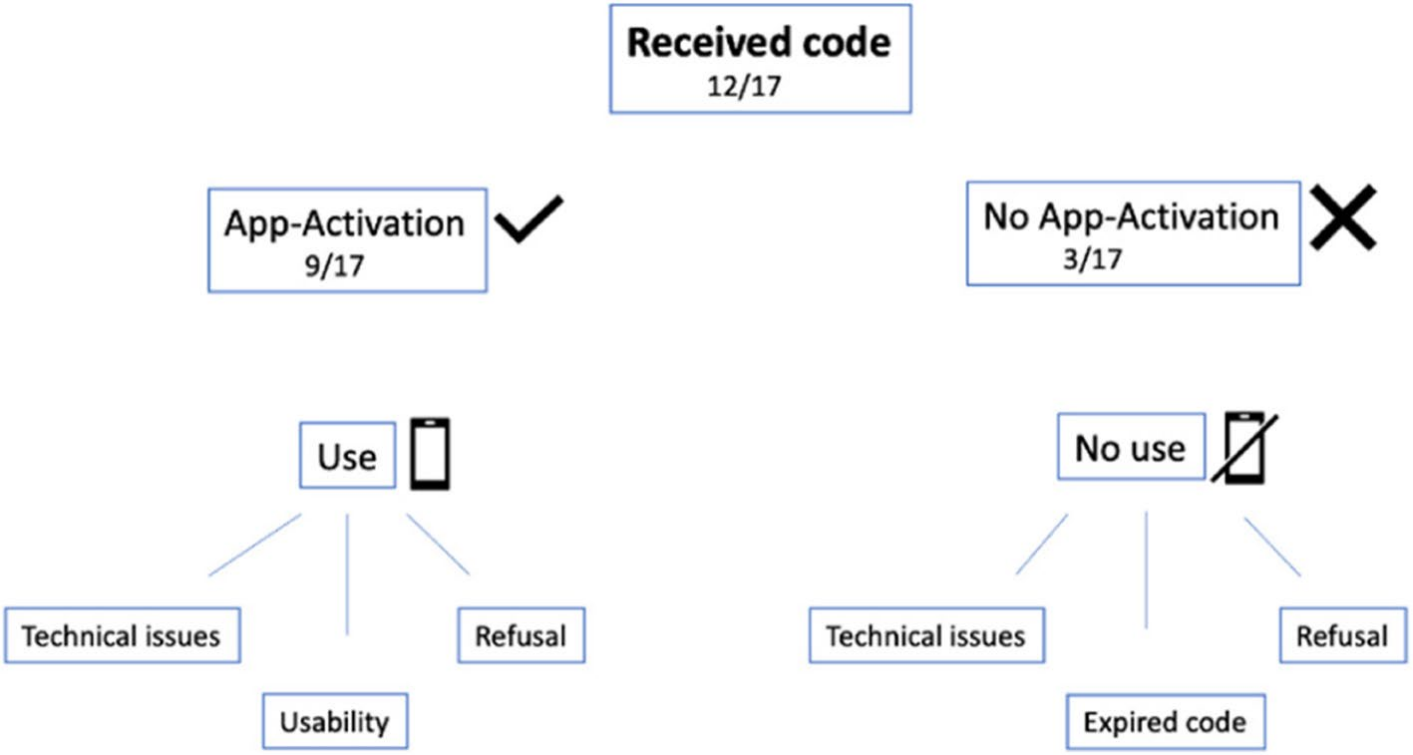
Development after code preservation using the hierarchical code subcode model. Source: Document analysis with MAXQDA

Driven by an interest in information about the lost-to-follow-ups of the main study participants, we were very interested in the redemption rate of prescriptions, activated accounts and the utilisation of Group 3. Figure 3 shows how varied the reasons for non-utilisation or participation are after receiving a prescription. One patient cancelled his participation in the study after receiving the prescription without giving reasons or information as to whether it was redeemed. A further 13 of the 17 patients surveyed had their prescriptions redeemed by the health insurance companies, 12 of whom received an access code. Only one patient did not receive a code from the health insurance company, despite correct transmission, on the grounds of being too old. As there is no age restriction, this is false information. Although 12 out of 17 successfully received a code, nine patients activated and used the digital health application ‘somnio’^®^ (Fig. 3).

### From code receipt onwards

#### No app-activation

Three patients gave the same reasons for non-activation after prescription redemption and code receipt as patients without prescription redemption: digital/technical skills in the absence of a contact person, progression of the disease or limited state of health with simultaneous expiry of the redemption period (expiry of prescription) or unclear instructions in addition to poor state of health.

*‘(…) I didn’t know what to do with the prescription…my health insurance company said you download apps from the App Store/Play Store. They are not responsible for that.’* (Patient 21, female, qualitative interview).

*‘(…) as my health was not very good for a long time*,* I unfortunately neglected your study completely. (…) The prescription then became invalid in the meantime.’* (Patient 24, male, qualitative interview).

#### App-activation and first experiences

A total of nine people dropped out of the main study despite using DiGA. The reasons for those patients who did not return at least two more completed questionnaires for the main study and are cases of lost-to-follow-up are discussed below.

One patient gave a detailed account of the technical difficulties he encountered after DiGA activation and initial user experience. These could not be resolved either with his technically skilled partner or with the help of the manufacturer’s support service.

‘(…) *at first I couldn’t use the app at all and after deleting and reinstalling it I could only use it up to the first lesson. I’m really fit in IT matters and even my wife - who works at the IT helpdesk of a large company - couldn’t get the app to work. The IT support was catastrophic or more precisely = 0. Only superficial mails*,* a phone call was refused.’* (Patient 23, male, qualitative interviews).

For a further two patients, the structure and interface design were not appealing enough to take part in the main study or even to continue using the digital health application ‘somnio’^®^.

*‘(…) the app does not meet my problem; the structure does not suit me; the application is too complicated to explain (voice control) and too many questions at the beginning.’ (Patient 27 and 19*,* female*,* qualitative interviews*).

After use, 2 patients lost interest in the programme and discontinued it for personal reasons:

*‘(…) As my session had recently ended*,* I did not request further participation for personal reasons. I ask for your understanding’ (Patient 25*,* female*,* qualitative interview*).

Three are satisfied with the use and were excluded due to their limited state of health, technical difficulties (after DiGA update) or due to a limited period of use of 90 days from the date of issue.

*‘(…) I’m in so much pain that I can’t concentrate on the app… but the structure is very nice. When my health improves*,* I can imagine using the app again.’* (Patient 13, female, qualitative interview).

*‘(…) the 1st module ran smoothly*,* nice little man*,* then the 2nd chapter was over*,* the app stopped loading.’* (Patient 16, female, qualitative interview).

*‘(…) because I didn’t manage to read through all the parts of the exercise(…)’* (Patient 22, male, qualitative interview).

#### Interest in follow-up prescription

In group 3, 6 patients expressed an interest in a follow-up prescription: 2 patients saw no need due to an improvement in their sleep disorder after completion of the cancer therapy, 1 patient felt that her state of health was so poor that she did not require a further prescription. 2 patients showed interest in a new prescription:

*‘(…) I would be very interested in it*,* as I am currently subject to very strong fluctuations in my sleep rhythm and general sleep.’* (Patient 22, male, qualitative interview).

Another 2 patients rated their technical understanding as such that they would consider using it in the future with appropriate support, but at the same time shied away from the administrative effort involved in obtaining a new access code. One patient did not respond.

*‘(…) I’m not sure*,* I’m not as fit as the younger generation*,* but with guidance and help for the first steps*,* I’m confident.’* (Patient 12, female, qualitative interview).

A total of 5 patients reported persistent sleep disturbance despite the use of various techniques. One patient refused a follow-up prescription because improvement was achieved with the help of medication.

*(…) with the help of my psychologist*,* who is a behavioural therapist*,* I am also having difficulties getting to grips with the issue at the moment.’* (Patient 22, male, qualitative interview).

## Discussion

Sleep disturbance at different times is a frequently mentioned symptom burden in cancer patients [35]. DiGA represents a low-threshold therapeutic measure with low risk for patients. Although digital health application-based interventions are now an integral part of healthcare research, the reliability of randomised controlled trials (RCTs) is limited and restricts the generalisation of digital intervention study results [36, 37]. At the same time, there are few publications on drop-outs and references to methodological limitations due to low case numbers, lack of blinding and short study measurement times [20]. Kernebeck et al. therefore call for a stronger integration of qualitative designs [38]. In the course of our main study, there were also limitations due to non-participation or drop-outs at different phases of the main ‘SOMNUS’-study. This circumstance led to additional problem-centred qualitative interviews on subjective experiences and difficulties before and after a DiGA prescription was issued. The different times and types of drop-outs were taken into account so that social behaviour could be uncovered and interpreted.

In all three groups, the highest frequency is no use of tranquillisers, antidepressants or sleeping tablets (see Additional file 4). This suggests that digital health applications may offer a realistic, low-risk alternative for this patient group. In alignment with the findings of the National Association of Statutory Health Insurance Funds, we also have observed a higher participation of women in group 3 compared to men [7]. However, given the limited number of cases in this qualitative study, it is challenging to draw any definitive conclusions regarding gender-specific differences. Nevertheless, our findings suggest that the same challenges are encountered by patients of both genders.

The decision of 177 patients to refuse participation altogether allows various assumptions to be made. Of note is the 62% of patients in group 1 who refused to participate, citing the subjective perception that they did not experience the sleep disorder. This raises the question of whether insomnia is a symptom that affects the quality of life of cancer patients. In this context, we would like to refer to the results of our previous research, which focused on suitable screening questions to identify insomnia in cancer patients. There it was shown that a question focused on tiredness is less effective in identifying insomnia in cancer patients. In contrast, a question about sleep disorder was found to be more suitable. So, the proposal of a sleep application as a possible treatment option, also for patients selected solely on the basis of tiredness, may have contributed to the frequent indication of this reason of refusal [39]. For future studies, it may be beneficial to prioritize patients who report sleep disorders alone. Screening instruments remain a valuable tool for identifying symptom burden, especially in cancer patients. Within our study, based on the screening values, doctors were sensitised to the issue, addressed it and the patients told their doctors in the medical history interview whether they were suffering from it. This procedure is in line with Dahiya et al.‘s conclusions that sleep disorders are often not addressed by cancer patients and therefore remain unrecognised. This is because with complex overlapping symptom burdens and time-limited doctor-patient contacts, cancer patients focus the conversation on the primary disease and may limit comprehensive therapeutic success [40, 41]. Systematic recording with screening instruments can help to broaden the view and open the doors for a conversation, especially since in group 1 only 3% (*n* = 177) cited a lack of technical equipment as a reason for refusal and therefore DiGA-capable devices are available or favour their use. In groups 1 and 2, concomitant illnesses or current therapies that have an influence on the quality of sleep were mentioned. For example, some patients in our study also experienced an improvement in sleep disturbances after completing radiotherapy and chemotherapy, as these could be identified as concomitant symptoms before and during treatment [16, 42].

Again, in group 2, there are cases where physicians decided not to prescribe, mainly because of deteriorating health or treatment-related symptom burden. Sleep disturbance caused by current treatment was also not considered to be present at follow-up consultations. In these cases, the reasons were due to cancer-related factors. So, medical assessment remains essential, particularly in the case of a dynamic course of the disease. A qualitative survey of French general practitioners also confirms our findings that diagnostic tasks and the comprehensive assessment of patients are the sole responsibility of physicians and cannot be replaced by digital tools [43].

The literature revealed that the barriers identified in our study are not exclusive to the oncological field [25]. They can be transferred to other patient groups as well. For example, patients with chronic obstructive pulmonary disease (COPD) have also reported technical issues when using a DiGA to promote physical activity. These problems could not be resolved immediately, which led to the discontinuation of the usage of a DiGA [44]. In our study this is especially the case when, for technical reasons, the correct contact details are missing, or no one feels responsible for the information.

The results in group 3 illustrate the complex reasons for the lost-to-follow-up at different points in time: after receiving the prescription, only a few patients justified non-utilisation with progressively reduced health status and/or failure to redeem the prescription within the redemption period. A follow-up prescription was generally rejected due to the additional administrative work involved and consequently led to the study being cancelled. The further detailed analyses show that several patients with advanced cancer stages and advanced age received a prescription, redeemed it and used the DiGA. Consequently, these reasons did not represent a primary reason for refusal. In the course of the additional survey, technical barriers in various formats emerged that further reduced the active users in group 3, especially since again no quick solution could be found [22, 45]. The results show that it is not clear to the patients whether the cancellation is due to their own digital skills or external conditions, such as a lack of simple instructions in simple/translated language and/or a lack of contact persons in the system. This group of patients also initially expressed interest in a second prescription and then declined due to the additional administrative work involved. This finding is also consistent with the DiGA report that only 10% of patients have follow-up certificates, regardless of whether they need them or not. Our data also suggests that patients with very advanced cancer in particular need low-threshold pathways for follow-up prescriptions or ongoing use authorisation procedures [7]. In a study on mobile applications for vulnerable groups, Sarkar et al. emphasise already 2016 the importance of user-friendliness to ensure widespread use by patients. Specifically, they call for more automated functions, less manual data entry and simple language supplemented by graphics [26]. A playful structure with easy-to-understand language and intuitive usage is also rated positively by Wangler et al. [46].

Accordingly, these findings are consistent with the results of our qualitative survey of group 3, where the structure of DiGA with contact person Albert was perceived as pleasant and only a minority did not like it. In this context, we agree with the position by Dittrich et al. that the inclusion of patient-reported outcomes is crucial to gain insight into usability [47]. This broad usability can be strengthened through easy access to prescriptions and easy-to-understand (multilingual) instructions for activating digital health applications and systematic training and and the appointment of a responsible contact person, i.e. digital assistance in the healthcare system.

An increase in the level of information available to the physicians may also help to support the uptake of DiGAs. A recent study conducted Dahlhausen et al. indicated that healthcare professionals have the greatest influence on patient adherence and play a significant role in the implementation process [48]. Nevertheless, at the same time, physicians do not feel sufficiently informed to counsel their patients on DiGAs [49]. It can thus be hypothesized that the expansion of informational opportunities for physicians and nursing staff could help reduce this barrier [46].

Some of the challenges faced by our study participants have been adapted by DiGA providers, particularly in light of the COVID-19 pandemic. For example, telephone accessibility has been improved, as has the option of easily accessible photo prescription redemption directly through the application’s website. The results of an online survey on patient acceptance of DiGAs demonstrated a considerable general willingness to employ them [50]. So, the removal of some barriers, together with existing patient acceptance and compliance, may promote successful use. Additional optimisation is addressed in the current DiGA report in the form of a ‘blended care concept’, a combination of personal treatment and digital intervention [8]. Further recommendations speak in favour of data exchange between DiGA providers and physicians [51]. If physicians can automatically chart the changes in symptom burden in their files, appropriate treatment plans can be implemented in accordance with the patient’s wishes.

This study shows that non-response analyses are an important part of primary studies and help to understand the different types of attrition. As this supports the validity and reliability of the main findings. In our case, keeping in touch and maintaining contact is an important part of panel studies.

In light to the findings, there are a few limitations to our study that are worth mentioning. The first limitation lies in the predefined inclusion criterion that only patients with an ISI score of 15 and above were included. It would be better to reduce this value to 8 for future studies. This is because it is assumed that cancer patients with an ISI of 8 can also benefit from DiGA.

In addition, high drop-out rates are to be expected, especially in DiGA trials. Small study sizes limit reliability and validity. This affects the representativity and generalisability of findings. In our case, we were interested in non-respondents, and for this purpose we received many responses regarding non-participation in our study; this applies to all three groups of non-participation. Therefore, the strength of this study lies in these high response rates, as we were able to track very precisely why patients were not eligible for DiGA or ended up in the lost to follow-up group. Interestingly, we had the same problem with low response rates in the intervention group that Kolominsky-Rabas et al. addressed in their study on the methodological quality of DiGAs [20]. The fact that participants in the intervention group used the DiGA without responding to our questionnaires suggests a different strategy.

This results in a structural problem within unfunded doctoral research at German universities. At the level of self-reflection, the results suggest that closer monitoring of patients could possibly reduce lost-to-follow-up in group 3, as research takes place alongside or at the periphery of the doctor’s working hours and time resources for research are therefore limited. Nevertheless, or perhaps precisely for this reason, it has been shown that individual points such as the duration of the DiGA from the day of prescription need to be better communicated. The same applies to the lack of contact persons for comprehensive problem solving. Especially when it is not a genuine technical problem of the DiGA provider or when barriers arise that cannot be solved with two contacts in 1st and 2nd level support.

## Conclusions

As a neutral, validated instrument, screening tools can be a starting point/door opener for communication on the topic of sleep disorders and the prescription of digital applications. To counteract the concern of a reduction in doctor-patient contact through DiGAs, Kuhn et al. emphasise that DiGAs can be consolidated as a complementary and supportive treatment option if the sleep disorder continues to be recorded in a structured manner or is actively recorded in a personal conversation [52]. This allows the adequacy and effectiveness to be assessed and DiGAs to be recognised as an opportunity to overcome sleep disorders. A reduction to the stage of illness, technical skills of the patient and/or age would be a misinterpretation. Rather, a small group of vulnerable patients with progressive cancer diseases need reliable contact persons and digital assistance for the successful use of DiGAs. There is also a need for improvement in the renewal of prescriptions.

## Supplementary Information

Supplementary Material 1.

Supplementary Material 2.

Supplementary Material 3.

Supplementary Material 4.

Supplementary Material 5.

## Data Availability

Data is available from the corresponding author on reasonable request.

## Abbreviations

DiGA: Digital health application (Digitale Gesundheitsanwendung)
DVG: German Digital Healthcare Act (Digitale-Versorgung-Gesetz)
BfArM: German Federal Ministry for Drugs and Medical Devices (Bundesinstitut für Arzneimittel und Medizinprodukte)
PECAN: Processus de Certification des Applications Numérique de Santé
SOMNUS-study: Benefit of SOMNio in cancer patients-analysis of qUality of life and phySical activity
MIDOS^2^: Minimal Documentation System^2^
PHQ-8: Patient-Health-Questionnaire
ISI: Insomnia Severity Index
SPSS: Statistical Package for Social Sciences
UICC: Union for International Cancer Control
RCTs: Randomised controlled trials
COPD: Chronic obstructive pulmonary disease

## References

[1] Stern AD, Brönneke J, Debatin JF, Hagen J, Matthies H, Patel S, et al. Advancing digital health applications: priorities for innovation in real-world evidence generation. Lancet Digit Health. 2022;4(3):e200–6. 35216754 10.1016/S2589-7500(21)00292-2

[2] Medizinprodukte BfAu. Wie funktioniert das Fast-Track Verfahren? Available from: https://www.diga.bfarm.de/de/diga-hersteller. Accessed 15 Jul 2024.

[3] Jorzig A, Sarangi F. Digitale-Versorgung-Gesetz. In: Jorzig A, Sarangi F, editors. Digitalisierung im Gesundheitswesen: Ein kompakter Streifzug durch Recht, Technik und Ethik. Springer, Berlin Heidelberg: Berlin, Heidelberg; 2020. p. 41–50.

[4] Mittermaier M, Sina C, Richter JG, Raspe M, Stais P, Vehreschild J, et al. Praktische Anwendung digitaler Gesundheitsanwendungen (DiGA) in der Inneren Medizin. Der Internist. 2022;63(3):245–54. 35037948 10.1007/s00108-022-01264-5

[5] Gesundheit Bf. 2024. Available from: https://www.bundesgesundheitsministerium.de/themen/krankenversicherung/online-ratgeber-krankenversicherung/arznei-heil-und-hilfsmittel/digitale-gesundheitsanwendungen#:~:text=Wie%20erhalte%20ich%20eine%20DiGA,Antragsfragen%20halten%20die%20Krankenkassen%20bereit. Accessed 24 Jul 2024.

[6] Rinsche F. The Role of Digital Health Care Startups. Crossing Borders- Innovation in the U.S. Health Care System: Schmid, A Singh, S; 2017.

[7] Stoff-Ahnis S. Bericht des GKV-Spitzenverbandes über die Inanspruchnahme und Entwicklung der Versorgung mit digitalen Gesundheitsanwendungen (DiGA-Bericht). 2022.

[8] Meskendahl D, Bachmann T. Marktentwicklung Digitaler Gesundheitsanwendungen (DiGA-Report). 2023. Available from: https://www.digitalversorgt.de/wp-content/uploads/2024/01/DiGA-Report-2023-SVDGV.pdf. Accessed 23 Jul 2024

[9] European Institute for Innovation and Technology-Health. European Taskforce for Harmonised Evaluations of Digital Medical Devices (DMDs). Available from: https://www.eithealth.eu/external-collaborations/european-taskforce-for-harmonised-evaluations-of-digital-medical-devices-dmds/. 2024

[10] Medizinprodukte BfAu. DiGA-Verzeichnis 2023. Available from: https://www.diga.bfarm.de/de/verzeichnis. Accessed 15 July 2024

[11] GmbH M. Alle Funktionen von somnio erklärt. Available from: https://www.somn.io/app/wie-funktioniert-somnio/. Accessed 15 July 2024

[12] Riemann D, Baglioni C, Bassetti C, Bjorvatn B, Dolenc Groselj L, Ellis JG, et al. European guideline for the diagnosis and treatment of insomnia. J Sleep Res. 2017;26(6):675–700. 28875581 10.1111/jsr.12594

[13] Lorenz N, Heim E, Roetger A, Birrer E, Maercker A. Randomized Controlled Trial to Test the Efficacy of an Unguided Online Intervention with Automated Feedback for the Treatment of Insomnia. Behav Cogn Psychother. 2019;47(3):287–302. 30185239 10.1017/S1352465818000486

[14] Al Maqbali M, Al Sinani M, Alsayed A, Gleason AM. Prevalence of Sleep Disturbance in Patients With Cancer: A Systematic Review and Meta-Analysis. Clin Nurs Res. 2022;31(6):1107–23. 35484919 10.1177/10547738221092146 PMC9266067

[15] Mercadante S, Valle A, Cartoni C, Pizzuto M. Insomnia in patients with advanced lung cancer admitted to palliative care services. Int J Clin Pract. 2021;75(10):e14521. 34120396 10.1111/ijcp.14521

[16] Savard J, Morin CM. Insomnia in the context of cancer: a review of a neglected problem. J Clin Oncol. 2001;19(3):895–908. 11157043 10.1200/JCO.2001.19.3.895

[17] Wu JQ, Appleman ER, Salazar RD, Ong JC. Cognitive Behavioral Therapy for Insomnia Comorbid With Psychiatric and Medical Conditions: A Meta-analysis. JAMA Int Med. 2015;175(9):1461–72. 10.1001/jamainternmed.2015.3006 26147487

[18] Geiger-Brown JM, Rogers VE, Liu W, Ludeman EM, Downton KD, Diaz-Abad M. Cognitive behavioral therapy in persons with comorbid insomnia: A meta-analysis. Sleep Med Rev. 2015;23:54–67. 25645130 10.1016/j.smrv.2014.11.007

[19] Labinsky H, Gupta L, Raimondo MG, Schett G, Knitza J. Real-world usage of digital health applications (DiGA) in rheumatology: results from a German patient survey. Rheumatol Int. 2023;43(4):713–9. 36543961 10.1007/s00296-022-05261-7 PMC9770561

[20] Kolominsky-Rabas PL, Tauscher M, Gerlach R, Perleth M. Dietzel N [How robust are studies of currently permanently included digital health applications (DiGA)? Methodological quality of studies demonstrating positive health care effects of DiGA]. Z Evid Fortbild Qual Gesundhwes. 2022;175:1–16. 36437182 10.1016/j.zefq.2022.09.008

[21] Baas J. DiGA-Report 2022. 2022:109-11. Available from: https://www.tk.de/resource/blob/2126090/778e6135918696524cccc1b7be39fd1b/diga-report-data.pdf. Accessed 29 Sept 2024.

[22] Jeffrey B, Bagala M, Creighton A, Leavey T, Nicholls S, Wood C, et al. Mobile phone applications and their use in the self-management of Type 2 Diabetes Mellitus: a qualitative study among app users and non-app users. Diabetol Metab Syndr. 2019;11:84. 31636719 10.1186/s13098-019-0480-4 PMC6794726

[23] Keum J, Chung MJ, Kim Y, Ko H, Sung MJ, Jo JH, et al. Usefulness of Smartphone Apps for Improving Nutritional Status of Pancreatic Cancer Patients: Randomized Controlled Trial. JMIR Mhealth Uhealth. 2021;9(8):e21088. 34463630 10.2196/21088 PMC8441607

[24] Nouri SS, Avila-Garcia P, Cemballi AG, Sarkar U, Aguilera A, Lyles CR. Assessing Mobile Phone Digital Literacy and Engagement in User-Centered Design in a Diverse, Safety-Net Population: Mixed Methods Study. JMIR Mhealth Uhealth. 2019;7(8):e14250. 31469083 10.2196/14250 PMC6740160

[25] Giebel GD, Speckemeier C, Abels C, Plescher F, Börchers K, Wasem J, et al. Problems and Barriers Related to the Use of Digital Health Applications: Scoping Review. J Med Internet Res. 2023;25:e43808. 37171838 10.2196/43808 PMC10221513

[26] Sarkar U, Gourley GI, Lyles CR, Tieu L, Clarity C, Newmark L, et al. Usability of Commercially Available Mobile Applications for Diverse Patients. J Gen Int Med. 2016;31(12):1417–26. 10.1007/s11606-016-3771-6 PMC5130945 27418347

[27] Dhar E, Bah AN, Chicchi Giglioli IA, Quer S, Fernandez-Luque L, Núñez-Benjumea FJ, et al. A Scoping Review and a Taxonomy to Assess the Impact of Mobile Apps on Cancer Care Management. Cancers (Basel). 2023;15(6). https://www.mdpi.com/2072-6694/15/6/1775. 10.3390/cancers15061775 PMC10046563 36980661

[28] Weitzel EC, Schwenke M, Schomerus G, Schönknecht P, Bleckwenn M, Mehnert-Theuerkauf A, et al. E-mental health in Germany - what is the current use and what are experiences of different types of health care providers for patients with mental illnesses? Arch Public Health. 2023;81(1):133. 37461064 10.1186/s13690-023-01150-y PMC10353209

[29] Anderson K, Burford O, Emmerton L. Mobile Health Apps to Facilitate Self-Care: A Qualitative Study of User Experiences. PLoS One. 2016;11(5):e0156164. 27214203 10.1371/journal.pone.0156164 PMC4876999

[30] Tewes M, Rettler TM, Beckmann M, Scheer K, Ritterbusch U, Schuler M, et al. Patient-Reported-Outcome-Messung (PROM) psychosozialer Belastung und Symptome für ambulante Patienten unter kurativer oder palliativer Tumortherapie. Die Onkologie. 2018;24(1):69–75.

[31] Stiel S, Matthes ME, Bertram L, Ostgathe C, Elsner F, Radbruch L. Validation of the new version of the minimal documentation system (MIDOS) for patients in palliative care : the German version of the edmonton symptom assessment scale (ESAS). Schmerz. 2010;24(6):596–604. 20882300 10.1007/s00482-010-0972-5

[32] Kroenke K, Strine TW, Spitzer RL, Williams JB, Berry JT, Mokdad AH. The PHQ-8 as a measure of current depression in the general population. J Affect Disord. 2009;114(1–3):163–73. 18752852 10.1016/j.jad.2008.06.026

[33] Dieck A, Morin CM, Backhaus J. A German version of the Insomnia Severity Index. Somnologie. 2018;22(1):27–35.

[34] Rädiker S, Kuckartz U. Intercoder-Übereinstimmung analysieren. In: Rädiker S, Kuckartz U, editors. Analyse qualitativer Daten mit MAXQDA: Text, Audio und Video. Wiesbaden: Springer Fachmedien Wiesbaden; 2019. p. 287–303.

[35] Büttner-Teleagă A, Kim YT, Osel T, Richter K. Sleep Disorders in Cancer-A Systematic Review. Int J Environ Res Public Health. 2021;18(21). https://www.mdpi.com/1660-4601/18/21/11696. 10.3390/ijerph182111696 PMC8583058 34770209

[36] Eikermann M. Digitale Gesundheitsanwendungen aus Sicht der evidenzbasierten Medizin. KVH Journal 2022. Available from: https://journal.kvhh.net/6-2022/digitale-gesundheitsanwendungen-aus-sicht-der-evidenzbasierten-medizin. Accessed 29 Sept 2024.

[37] Lantzsch H, Eckhardt H, Campione A, Busse R, Henschke C. Digital health applications and the fast-track pathway to public health coverage in Germany: challenges and opportunities based on first results. BMC Health Serv Res. 2022;22(1):1182. 36131288 10.1186/s12913-022-08500-6 PMC9490912

[38] Kernebeck S, Scheibe M, Sinha M, Fischer F, Knapp A, Timpel P, et al. Development, Evaluation and Implementation of Digital Health Interventions (Part 1) - Discussion Paper of the Digital Health Working Group of the German Network for Health Services Research (DNVF). Gesundheitswesen. 2023;85(1):58–64. 36446615 10.1055/a-1933-2779 PMC11248393

[39] Pfeifer K, Ates G, Pogorzelski M, Zaun G, Rötger A, Schuler M, et al. Investigation of screening questions to identify insomnia in cancer patients. Sci Rep. 2024;14(1):18343. 39112537 10.1038/s41598-024-69086-z PMC11306326

[40] Leitlinienprogramm Onkologie (Deutsche Krebsgesellschaft, Deutsche Krebshilfe, AWMF): Palliativmedizin für Patienten mit einer nicht- heilbaren Krebserkrankung, Langversion. 2020.

[41] Dahiya S, Ahluwalia MS, Walia HK. Sleep disturbances in cancer patients: underrecognized and undertreated. Cleve Clin J Med. 2013;80(11):722–32. 24186891 10.3949/ccjm.80a.12170

[42] Rades D, Kopelke S, Schild SE, Kjaer TW, Tvilsted S, Bartscht T. Evaluation of Sleep Disturbances in Patients With Bladder Cancer Scheduled for Local or Loco-regional Radiochemotherapy. Anticancer Res. 2022;42(9):4511–5. 36039419 10.21873/anticanres.15953

[43] Sarradon-Eck A, Bouchez T, Auroy L, Schuers M, Darmon D. Attitudes of General Practitioners Toward Prescription of Mobile Health Apps: Qualitative Study. JMIR Mhealth Uhealth. 2021;9(3):e21795. 33661123 10.2196/21795 PMC7974757

[44] Bentley CL, Powell L, Potter S, Parker J, Mountain GA, Bartlett YK, et al. The Use of a Smartphone App and an Activity Tracker to Promote Physical Activity in the Management of Chronic Obstructive Pulmonary Disease: Randomized Controlled Feasibility Study. JMIR Mhealth Uhealth. 2020;8(6):e16203. 32490838 10.2196/16203 PMC7301262

[45] Torbjørnsen A, Ribu L, Rønnevig M, Grøttland A, Helseth S. Users’ acceptability of a mobile application for persons with type 2 diabetes: a qualitative study. BMC Health Serv Res. 2019;19(1):641. 31492176 10.1186/s12913-019-4486-2 PMC6729081

[46] Wangler J, Jansky M. Digitale Gesundheitsanwendungen (DiGA) in der Primärversorgung – Erfahrungen und Beobachtungen von Hausärzt*innen hinsichtlich der Anwendung von DiGA. Prävention und Gesundheitsförderung. 2023;18(4):483–91.

[47] Dittrich F, Mielitz A, Pustozerov E, Lawin D, von Jan U, Albrecht UV. Digital health applications from a government-regulated directory of reimbursable health apps in Germany-a systematic review for evidence and bias. Mhealth. 2023;9:35. 38023782 10.21037/mhealth-23-17 PMC10643174

[48] Dahlhausen F, Zinner M, Bieske L, Ehlers JP, Boehme P, Fehring L. There’s an app for that, but nobody’s using it: Insights on improving patient access and adherence to digital therapeutics in Germany. Digit Health. 2022;8:20552076221104670. 35811758 10.1177/20552076221104672 PMC9260569

[49] Dahlhausen F, Zinner M, Bieske L, Ehlers JP, Boehme P, Fehring L. Physicians’ Attitudes Toward Prescribable mHealth Apps and Implications for Adoption in Germany: Mixed Methods Study. JMIR Mhealth Uhealth. 2021;9(11):e33012. 34817385 10.2196/33012 PMC8663495

[50] Uncovska M, Freitag B, Meister S, Fehring L. Patient Acceptance of Prescribed and Fully Reimbursed mHealth Apps in Germany: An UTAUT2-based Online Survey Study. J Med Syst. 2023;47(1):14. 36705853 10.1007/s10916-023-01910-x PMC9880914

[51] Knitza J, Simon D, Lambrecht A, Raab C, Tascilar K, Hagen M, et al. Mobile Health Usage, Preferences, Barriers, and eHealth Literacy in Rheumatology: Patient Survey Study. JMIR Mhealth Uhealth. 2020;8(8):e19661. 32678796 10.2196/19661 PMC7450373

[52] Kuhn E, Rogge A, Schreyer K, Buyx A. Apps on Prescription in the Medical Office, but how? A Case-based Problem Outline of Medical-ethical Implications of DHA Usage. Gesundheitswesen. 2022;84(8–09):696–700. 33957698 10.1055/a-1473-5655

