# Supplementary material for: Applicability of a digital health application for cancer patients: a qualitative non-participation analysis
Source: BMC Health Serv Res. 2024 Oct 5;24:1187. doi: 10.1186/s12913-024-11654-0 (PMC11453002; doi:10.1186/s12913-024-11654-0)
Supplement: Supplementary file 1 — Supplementary Material 1. [file 12913_2024_11654_MOESM1_ESM.docx]

**Problem-centred interview guide for a Non-Participation Analysis**

First of all, thank you for taking the time to speak with me today. My name is Klara Pfeifer, and I am supervising this study under the direction of PD Dr. Mitra Tewes.

We are pleased to hear that you are interested in exploring the potential of a digital application to address the sleep disorder you mentioned. You have received a prescription for this, and we have realized that we do not yet have any information about whether you are using the application to improve your sleep quality.

We would therefore be very grateful for your input to help us to understand the potential obstacles you may be facing. We would be delighted to work with you to find these out together.

Firstly, I would like to go back to when you received the prescription from your physician.

I have a few questions for you what happened next:

*What did you do with the prescription after your appointment?*

*Did you put it aside because other problems were more urgent?*

*Did the prescription expire in the meantime? (after 4 weeks of prescription)*

*Would you be interested in a further prescription?*

*Should the prescription and instructions be given to you with a more detailed explanation?*

*Did you know how to submit the prescription? (not at the pharmacy, but directly at the health insurance company´s office, send it by post, submit it via the health insurance company´s website or app)*

*Could you describe the subsequent events following the submission of the prescription?*

*Option: The health insurance company has not yet got back to me/ I have not yet received an activation code/ they have refused my prescription*

*Option: I received the prescription from my health insurance company, but then had problems with the installation/registration -> Can you describe these problems? For example, were you unable to find a button?*

*Option: I received the prescription code, was able to install the app on an end device and then had technical problems using the individual modules. -> Can you describe these technical problems?*

*Option: I was able to complete the modules, but had difficulties accepting the app (filling in the sleep diary regulary, reducing bedtime, relaxation exercises…)*

*Option: The application was too stressful alongside my therapy.*

*There is another reason „...“*

Finally: *Do you see a benefit for yourself? Or have you found a different way to manage your sleep disorder?*

Thank you so much for sharing your experiences with us.
