# Supplementary material for: Applicability of a digital health application for cancer patients: a qualitative non-participation analysis
Source: BMC Health Serv Res. 2024 Oct 5;24:1187. doi: 10.1186/s12913-024-11654-0 (PMC11453002; doi:10.1186/s12913-024-11654-0)
Supplement: Supplementary file 2 — Supplementary Material 2. [file 12913_2024_11654_MOESM2_ESM.docx]

Additional file 1: *Patient characteristics - Group 1*


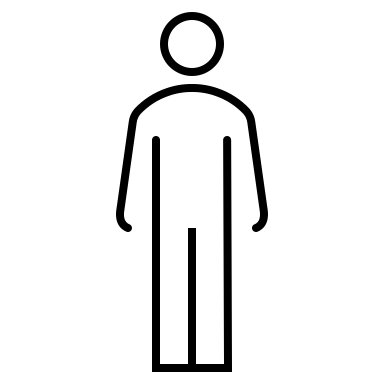


**55%**


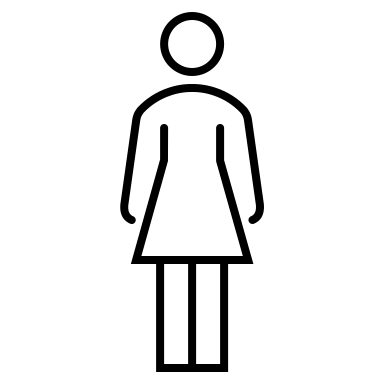


**45%**

Sex: n=177


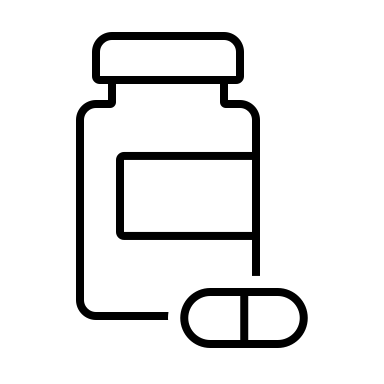

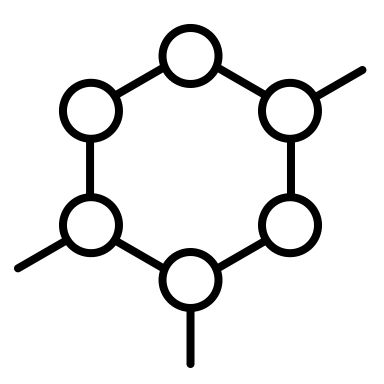

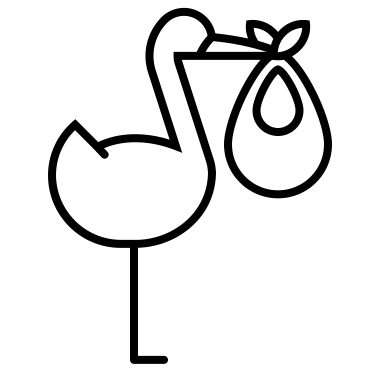


Age: Mean (Std.dev.)

**61 years (±12)**

Cancer stages: UICC n= 168


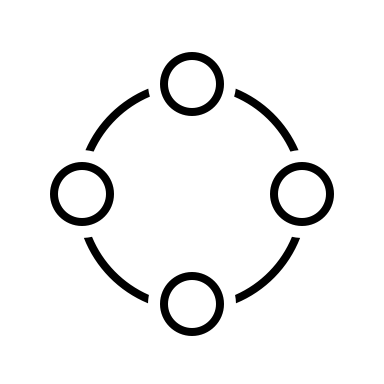


**I: 6%**

**IV: 80%**

**II: 3%**

**III: 11%**

No: **75%-**

Occasionally: **8%**

Daily: **17%**

Lung: **38%**

Gastrointestinal**: 23%**

Sarcoma: **10%**

Urinary tract: **11%**

Breast: **5%**

Other: **13%**

Sleeping pills/antidepressants: n= 171

Cancer entity: n= 177

Source: Document analysis; reporting period: Jan. 2021-May 2022; own calculations
