# Supplementary material for: Applicability of a digital health application for cancer patients: a qualitative non-participation analysis
Source: BMC Health Serv Res. 2024 Oct 5;24:1187. doi: 10.1186/s12913-024-11654-0 (PMC11453002; doi:10.1186/s12913-024-11654-0)
Supplement: Supplementary file 3 — Supplementary Material 3. [file 12913_2024_11654_MOESM3_ESM.docx]

Additional file 2: *Patient characteristics - Group 2*


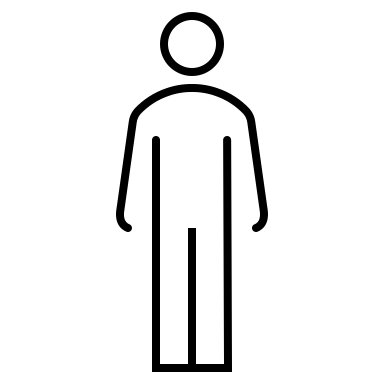


**9**


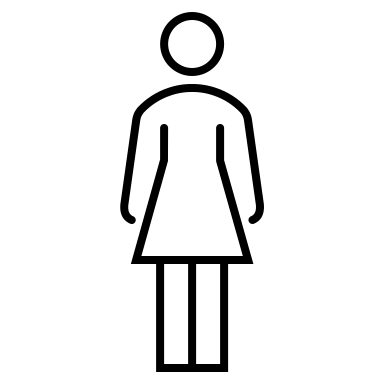


**3**

Sex: n=11


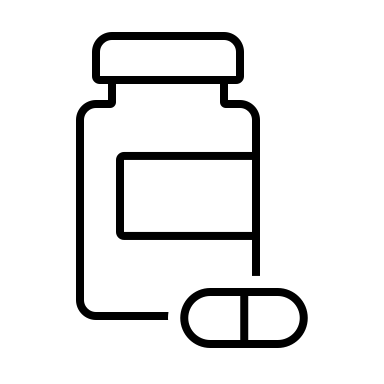

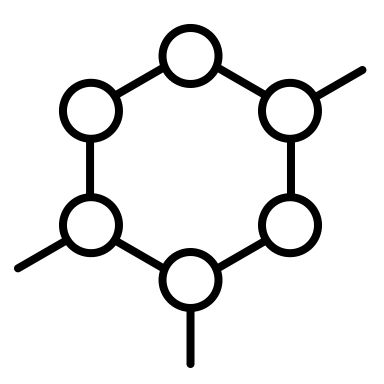

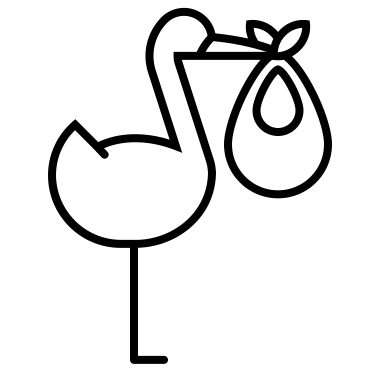


Age: median (range)

**58 years (42)**

Cancer stages: UICC n= 11


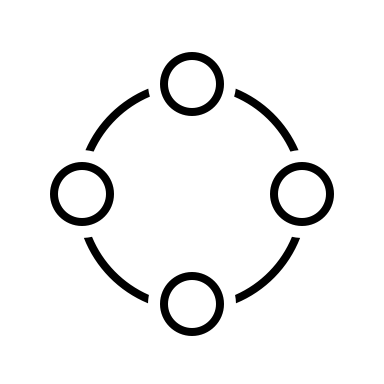


**I: 1**

**IV: 10**

**II: 0**

**III: 0**

No: **5**

Occasionally: **3**

Daily: **2**

Lung: **2**

Gastrointestinal: **3**

Sarcoma: **1**

Urinary tract: **2**

Breast: **1**

Other: **2**

Sleeping pills/antidepressants: n= 10

Cancer entity: n= 11
