# Supplementary material for: Applicability of a digital health application for cancer patients: a qualitative non-participation analysis
Source: BMC Health Serv Res. 2024 Oct 5;24:1187. doi: 10.1186/s12913-024-11654-0 (PMC11453002; doi:10.1186/s12913-024-11654-0)
Supplement: Supplementary file 4 — Supplementary Material 4. [file 12913_2024_11654_MOESM4_ESM.docx]

Additional file 4: Patient characteristic – Group 1-3

| **Characteristic** |  | Group 1 (n=177) | Group 2 (n=11) | Group 3 (n=17) |
| --- | --- | --- | --- | --- |
|  |  |  |  |  |
| Mean age/Std.dev. | | 61/12 | 58/11 | 58/9 |
| Sex n (%) |  |  |  |  |
| male |  | 98 (55%) | 9 | 6 |
| female |  | 79 (45%) | 2 | 11 |
| Cancer Entity n (%) | |  |  |  |
| Lung Cancer |  | 67 (38%) | 2 | 4 |
| Gastrointestinal Cancer | | 41 (23%) | 3 | 1 |
| Sarcoma |  | 18 (10%) | 1 | 2 |
| Urinary tract |  | 19 (11%) | 2 | 1 |
| Breast Cancer |  | 9 (5%) | 1 | 4 |
| Other |  | 23 (13%) | 2 | 5 |
| n |  | 177 | 11 | 17 |
| missing |  | 0 | 0 | 0 |
| Cancer Stages (UICC) n(%) | |  |  |  |
| I |  | 10 (6%) | 1 | 0 |
| II |  | 5 (3%) | 0 | 2 |
| III |  | 18(11%) | 0 | 2 |
| IV |  | 135 (80%) | 10 | 13 |
| n |  | 168 (100%) | 11 | 17 |
| missing |  | 9 | 0 | 0 |
| Taking sleeping pills, antidepressants n (%) | | |  |  |
| no |  | 129 (75%) | 5 | 9 |
| occasionally |  | 13 (8%) | 3 | 1 |
| daily |  | 29 (17%) | 2 | 6 |
| n |  | 171 | 10 | 16 |
| missing |  | 6 | 1 | 1 |

Source: Document analysis; reporting period: Jan. 2021-May 2022; own calculations
