# Supplementary material for: Applicability of a digital health application for cancer patients: a qualitative non-participation analysis
Source: BMC Health Serv Res. 2024 Oct 5;24:1187. doi: 10.1186/s12913-024-11654-0 (PMC11453002; doi:10.1186/s12913-024-11654-0)
Supplement: Supplementary file 5 — Supplementary Material 5. [file 12913_2024_11654_MOESM5_ESM.docx]

Additional file 3:

Coding tree of the qualitative data from the prescription of the DiGA and the further utilization after receiving a prescription

Source: Document analysis with MAXQDA

No feedback

Improvement insomnia

Administrative effort

Only German language

Missing contact person

Technical issues

Limited period of use

Missing instructions

Problems with app modules

Dropout personal reasons

**Withdrawal of study participation**

**Interest in follow- up prescription**

Yes

State of health

No

State of health

Negative

State of health

Positive

**Experiences and usability**

Yes

State of health

Expired code

Technical issues

No

False information by health insurance company

**App-Activation**

Yes

No

**Code receiveed**

Yes

Missing instructions

Expiry of prescription

No

**Redemption at health insurance company**
